# Design and optimization of a polarization-insensitive Ti/TiO_2_ metamaterial absorber using particle swarm optimization for broadband solar–thermal applications

**DOI:** 10.1038/s41598-025-31966-3

**Published:** 2025-12-15

**Authors:** Mohammed R. Saeed, Saif H. Abdulwahid, Tara Afra, Monia Ferchichi, Mir Hamid Rezaei

**Affiliations:** 1 Department of Electrical Power Engineering, Al-Hussain University College, Kerbala, Iraq; 2 Najaf Technical Institute, Al-Furat Al-Awsat Technical University, Najaf, Iraq; 3 Department of Media Technology and Communications Engineering, Al-Hussain University College, Kerbala, Iraq; 4 Department of Electrical and Information Engineering, Polytechnic University of Bari, Bari, Italy; 5 Department of Management Information Systems, Applied College, University of Ha’il, P.O.Box 2440, Ha’il, Saudi Arabia; 6 Department of Communications and Electronics, School of Electrical and Computer Engineering, Shiraz University, Shiraz, Iran

**Keywords:** Metamaterial absorber, Polarization independency, Angle insensitivity, Solar absorber, Particle swarm optimization algorithm, Engineering, Materials science, Nanoscience and technology, Optics and photonics, Physics

## Abstract

In this paper, a polarization-insensitive and ultra-broadband metamaterial absorber composed of titanium (Ti) and titanium dioxide (TiO_2_) resonators is proposed. The multilayer architecture integrates three square resonators and one disk resonator, which are optimized to maximize absorption across the 0.25–4 μm spectral range. Particle swarm optimization (PSO) yields an average absorption of 98.99% and a bandwidth of 3533 nm (467–4000 nm). The structure maintains absorption above 80% for transverse magnetic (TM) polarization and above 90% for transverse electric (TE) polarization for wavelengths above 600 nm, even at incidence angles up to 60°. Under the Air Mass 1.5 Global spectrum (AM 1.5G) solar spectrum, the device achieves a solar absorption efficiency of 98.17% and a thermal emission efficiency of 99.26% at 1800 K. This performance is a result of the combined effects of surface plasmon polaritons, magnetic plasmons, and localized surface plasmon resonances. The absorber further demonstrates robustness against structural variations, maintaining over 90% absorption across the wavelength range of 250–4919 nm (BW = 4669 nm). The designed absorber exhibits a compact electrical size, with a total thickness of approximately 0.2λ and periodicity of 0.1λ, calculated at the longest operating wavelength (4 μm), confirming its subwavelength characteristics and suitability for planar integration. In comparison to existing Ti-based absorbers, this design provides both the broadest operational bandwidth and the highest efficiency, indicating strong potential for solar energy harvesting applications.

**Supplementary Information:**

The online version contains supplementary material available at 10.1038/s41598-025-31966-3.

## Introduction

Metamaterial absorbers have emerged as a transformative technology in photonics and materials science, offering advanced capabilities for controlling electromagnetic interactions at subwavelength scales^1–3^. These engineered structures enable optical responses unattainable in natural materials^4,5^. The unique properties of metamaterial absorbers, especially their ability to achieve near-perfect absorption across specific spectral ranges, have made them vital for applications ranging from photovoltaics and thermal photonics to biochemical sensing and stealth technologies^6–10^.

The fundamental challenge in designing high-performance broadband absorbers lies in simultaneously achieving several key characteristics: wide operational bandwidth, polarization insensitivity, and angular stability^11–13^. Conventional absorber designs based on natural materials often suffer from inherent bandwidth and angular response limitations due to their fixed dielectric properties. These limitations have spurred significant research into hybrid metal-dielectric metamaterials that can support multiple resonant modes across broad spectral ranges^14,15^. Among various material systems, titanium (Ti) and titanium dioxide (TiO_2_) have emerged as particularly promising candidates due to their outstanding optical tunability, chemical stability, and compatibility with standard nanofabrication processes^16–18^.

Recent advances in computational optimization techniques have further enhanced the design capabilities for metamaterial absorbers^19,20^. The particle swarm optimization (PSO) algorithm has proven particularly effective for solving complex, multi-parameter optimization problems in photonic design^21^. This bio-inspired algorithm mimics the collective behavior of swarms to efficiently explore high-dimensional parameter spaces^22^, making it ideal for optimizing intricate metamaterial structures^23^. When combined with full-wave electromagnetic simulations, PSO enables the discovery of non-intuitive geometries that maximize absorption performance^24^.

Recent studies have proposed a variety of broadband absorber configurations, yet several technical challenges persist. Liu et al.^25^. introduced a Ti-SiO_2_ absorber that achieved over 90% absorption across a 1555 nm bandwidth (421–1976 nm). However, its performance was highly dependent on polarization state and angle of incidence. Building on this design, Huang and Wang^26^expanded the absorption bandwidth to 1576 nm (329–1905 nm), but the device incorporated intricate geometrical features that complicated fabrication. Similarly, Gao et al.^27^. developed a refractory absorber with a 1650 nm bandwidth, though its efficiency dropped markedly beyond 2200 nm, restricting its use in the mid-infrared region. Mokhtari et al.^28^. employed an optimization algorithm to design an ultra-broadband absorber based on TiN and TiO_2_materials, but the reliance on complex resonator geometries posed significant challenges for fabrication using standard lithographic methods. More recently, Ehsanikachosang et al.^29^. demonstrated a Ti/TiO_2_absorber with an extended bandwidth of 3000 nm (200–3200 nm). However, the structure exhibited limited tolerance to fabrication imperfections. Also, Rezaei et al.^30^. reported a polarization‑independent and wide‑angle Ti-based metamaterial absorber with 3509 nm over 90% absorption bandwidth.

Traditional metamaterial absorbers often suffer from intrinsic trade-offs between bandwidth, angular tolerance, and polarization independence. Single-resonance plasmonic structures, such as cross-shaped or circular patch absorbers, typically exhibit high absorption only within a narrow spectral window^1,31^. Multilayer or gradient-index designs have expanded the bandwidth but still remain limited by destructive interference and impedance mismatch under oblique incidence^32^. These constraints hinder practical applications such as solar-thermal energy harvesting, thermophotovoltaics, and infrared detection, which require broadband, angle- and polarization-robust absorption for efficient light-to-heat conversion. Despite these advances, current Ti-based absorbers still face significant limitations. Although several studies have demonstrated broadband absorption using Ti metal, these designs typically exhibit limited bandwidth (typically < 2 μm) or reduced performance under oblique incidence. In addition, many existing absorbers show strong polarization dependence at large angles of incidence, restricting their practical utility^25,27,32–34^. These limitations highlight the need for new design paradigms that can simultaneously address bandwidth, angular stability, and polarization independence.

In this work, we overcome these challenges by employing PSO to design a multilayer Ti/TiO_2_ resonator structure that supports multiple hybridized plasmonic and cavity modes. This coupling mechanism yields ultra-wideband absorption exceeding 4000 nm, maintained across a broad angular range and for all polarizations. Such wideband absorption enhances solar–thermal conversion efficiency, increases spectral selectivity for thermal emitters, and reduces radiative losses, thereby improving overall device performance in high-temperature and energy-harvesting systems. Our approach combines three key innovations: (1) a hierarchical arrangement of square and disk resonators that collectively excite multiple resonant modes, (2) a systematic optimization framework using the PSO algorithm to achieve the optimal performance, and (3) a robust geometric configuration that maintains high efficiency despite fabrication variations. The structure exhibits remarkable absorption efficiency, with an average absorption (A_avg_) of 98.94% spanning the broad spectrum from 0.25 to 4 μm and absorbing over 92% of the incident wave across the entire studied wavelength range. It maintains consistent optical performance for varying polarization states up to an incident angle of 50° and retains absorption above 80% even at 60° incidence. The scientific significance of this work extends beyond its performance metrics.

The innovation of this work lies in achieving ultra-wideband absorption extending beyond 4 μm through optimized Ti/TiO_2_multilayer resonators. Extending the spectrum into the mid-infrared region enhances solar–thermal harvesting by capturing additional radiative energy, improving photothermal conversion and thermophotovoltaic efficiency in hot, high-irradiance environments, and enhancing total absorbed power and thermal stability for high-temperature operation. This performance arises from synergistic coupling among multiple resonant modes, including surface plasmon polaritons (SPPs), magnetic plasmons (MPs), localized surface plasmon resonances (LSPRs), and cavity resonances (CRs), that collectively yield a continuous broadband response. The approach aligns with recent advances in multi-mode hybridization strategies reported in Refs^35–39^.. Our findings challenge conventional design paradigms by demonstrating how carefully engineered coupling between these modes can create synergistic effects that enhance absorption bandwidth without compromising angular stability. From a practical perspective, the 98.17% solar absorption under AM 1.5G illumination and the robustness to fabrication tolerances make the proposed absorber particularly attractive for renewable energy applications.

The rest of this paper is organized as follows: Sect. 2 introduces the proposed absorber and outlines the fundamental principles of absorption. Section 3 presents the optimization methodology, showcasing the optimal geometric values obtained through the PSO algorithm. This section also examines various factors affecting the performance of the absorber, including types of resonances involved in the absorption process, the polarization and angle of the incident light, operating as a solar absorber, the influence of the presence of each resonator, and potential fabrication errors on the absorption behavior of the absorber. Finally, Sect. 4 expresses the conclusion.

### Schematic of the metamaterial absorber

**Fig. 1:**
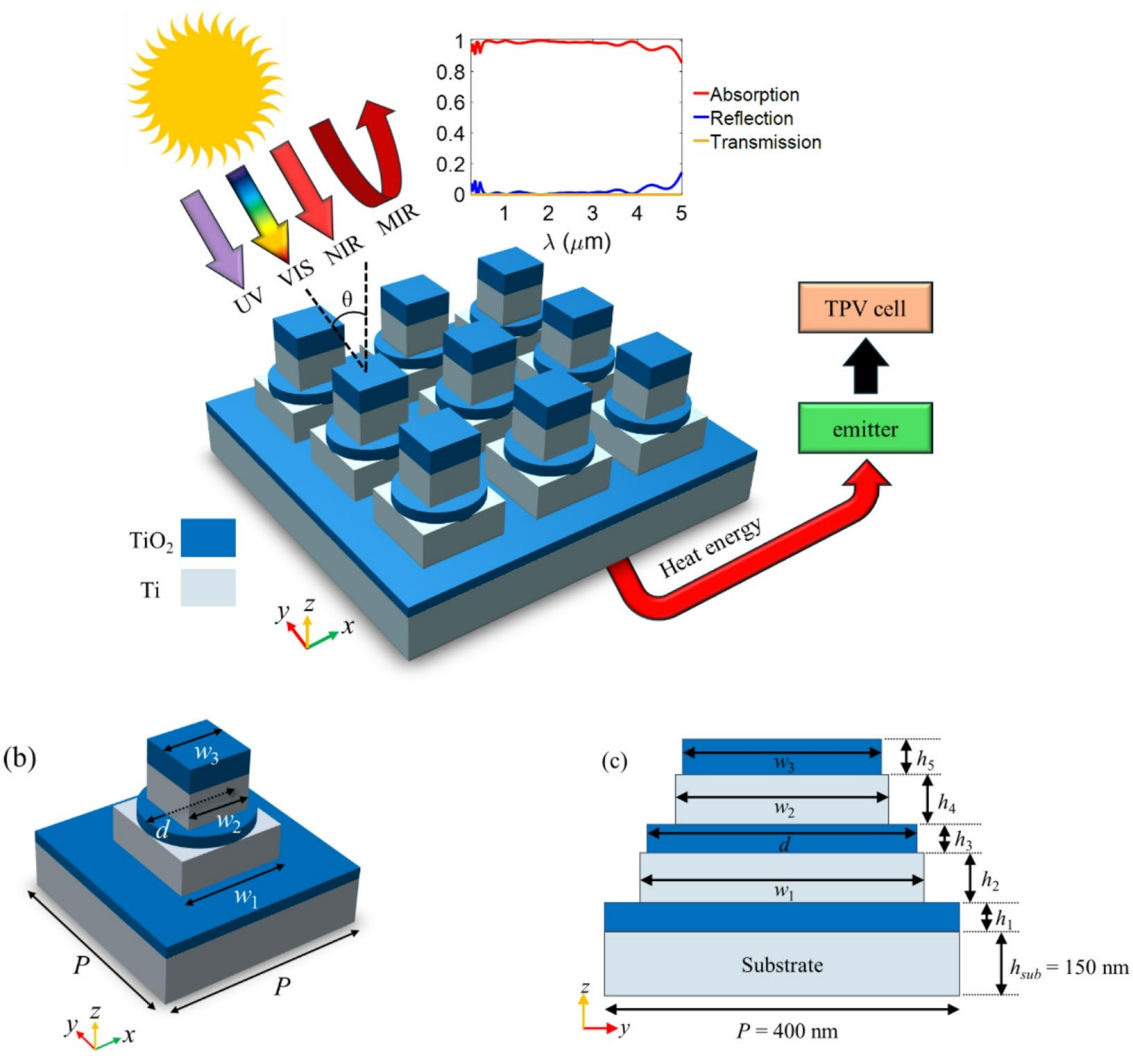
3D schematic of the (**a**) proposed absorber and (**b**) a unit cell. (**c**) Cross-sectional view (*y*-*z* plane) of the unit cell and the geometric variables.

Figure 1 illustrates the schematic of the proposed multilayer absorber. Figure 1(a) presents a three-dimensional (3D) representation of the absorber, showcasing its periodic arrangement of unit cells. Figure 1(b) provides a close-up view of the detailed geometry of a single unit cell, highlighting the layered composition that facilitates plasmonic resonance and enhances absorption. The unit cell consists of three square resonators, one disk resonator array, and a thick Ti substrate separated by a TiO_2_ layer. This structure, composed of several metallic and dielectric layers, forms a multilayer configuration. A cross-sectional view of the unit cell along the *y*-*z* plane is shown in Fig. 1(c), revealing key geometric variables such as the widths of the square resonators (*w*_1_, *w*_2_, and *w*_3_), the diameter of the disk resonator (*d*), the height of the TiO_2_ thin film (*h*_1_), and the heights of the resonators (*h*_2_, *h*_3_, *h*_4_, and *h*_5_). The periodicity of the unit cell (*P*) is 400 nm. The thickness of the Ti substrate layer is set to 150 nm, ensuring that no light is transmitted through this layer.

**Fig. 2:**
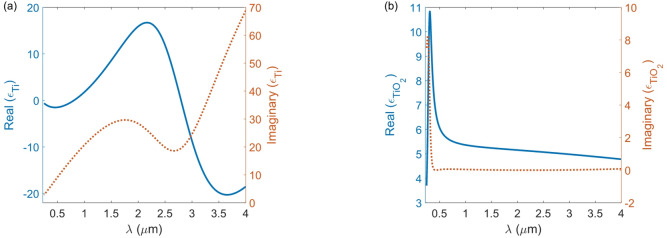
The real and imaginary components of the permittivities for (**a**) Ti and (**b**) TiO_2_.

The 3D finite-difference time-domain (3D-FDTD) method, using the commercial software Ansys Lumerical version 2020^40^, was used to evaluate the performance of the absorber. The permittivities (*ε*) of titanium Ti and TiO_2_ were obtained from the model established by Lide, commonly known as the CRC model^41^and from the research conducted by Siefke et al.^42,43^., which is illustrated in Fig. 2.

Given the periodic nature of the structure, only one unit cell has been simulated. To optimize computational efficiency, anti-symmetric and symmetric boundary conditions have been utilized in the *x* and *y* directions, respectively. At the same time, perfectly matched layers (PMLs) have been applied in the *z* direction. The simulation mesh sizes have been uniformly set to 5 nm across all spatial dimensions (*x*, *y*, and *z*) to ensure accuracy in the results. In addition, the incident light has been modeled using a plane-wave source with a wavelength range of 0.25–4 μm positioned at the top of the structure, propagating in the -*z* direction.

When an electromagnetic wave encounters an absorber, some part of the wave is reflected, another part penetrates the material, and the rest is absorbed. Equation (1) represents the absorption of an absorber in terms of its reflection and transmission as follows^30^:

1 $$A(\lambda )=1 - R(\lambda ) - T(\lambda )$$

where *A*(λ), *R*(λ), and *T*(λ) are the absorption, reflection, and transmission of the absorber, respectively. Generally, lower reflection and transmission correlate with higher absorption. Using a metal substrate of appropriate thickness can effectively bring transmission to zero. This required thickness is determined by a parameter known as skin depth (*δ*), which allows us to express the condition *T*(λ) = 0. The skin depth is calculated by the following Eq.^4^:

2 $$\delta =\sqrt {\frac{{\rho \lambda }}{{\pi c{\mu _0}{\mu _r}}}}$$

where *ρ* signifies the resistivity of the metal (Ω-m), λ denotes the wavelength (m), *c* represents the speed of light in a vacuum (m/s), *µ*_0_ is the permeability of free space (H/m) with a value of 4π × 10^− 7^, and *µ*_*r*_ is approximately equal to 1 for the metal. For Ti metal, the resistivity is *ρ* = 4.2 × 10^− 7^ Ω-m^45^, leading to minimum and maximum skin depths of *δ*_min_ = 9.4 nm and *δ*_max_ = 37.7 nm for wavelengths of 0.25 μm and 4 μm, respectively. Consequently, since the substrate thickness exceeds the maximum skin depth, the transmission for the proposed absorber is effectively zero.

### Achieving optimized geometric variables

**Fig. 3:**
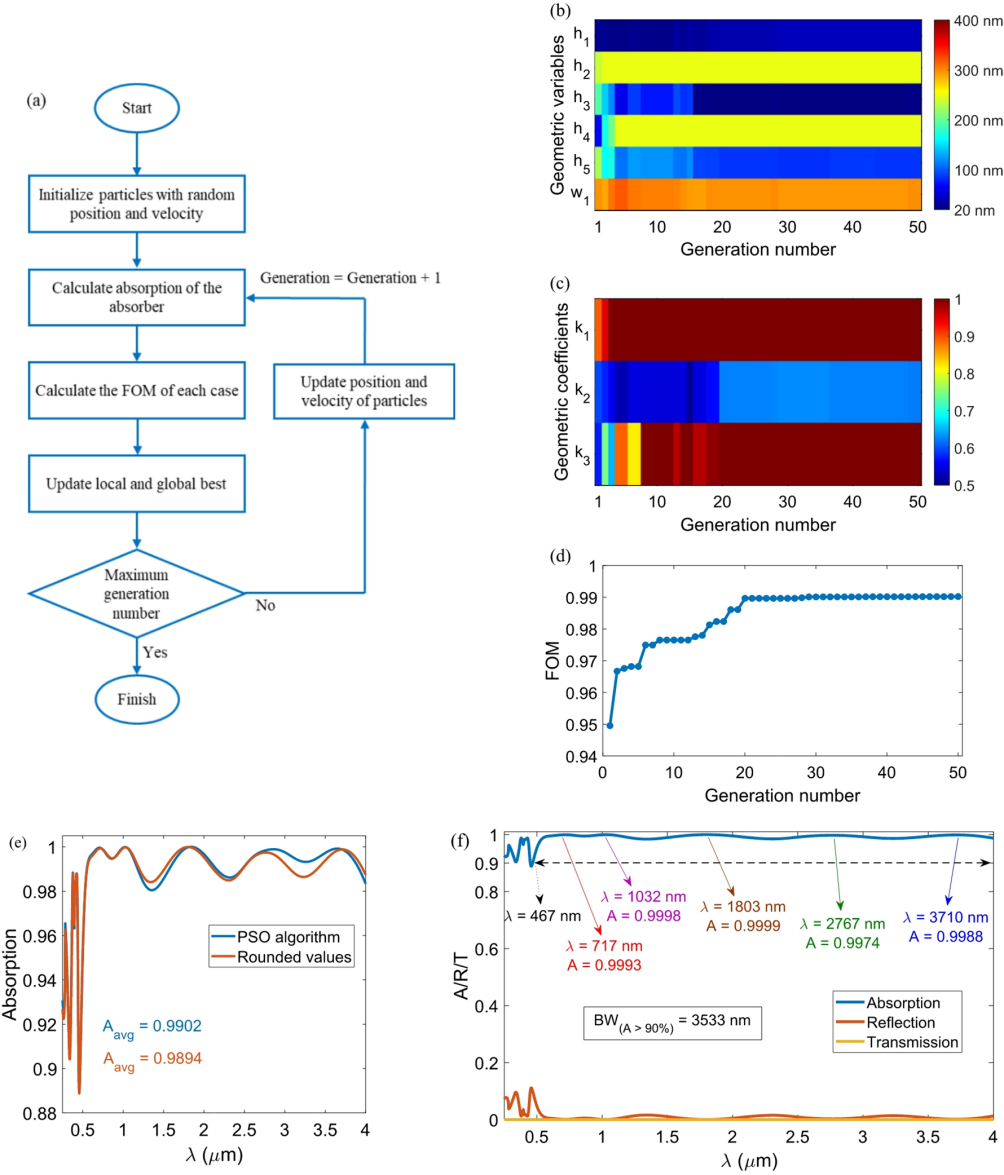
(**a**) Flowchart of the PSO algorithm. Change in (**b**) geometric variables and (**c**) geometric coefficients with generation number. (**d**) FOM of the absorber as a function of generation number. (**e**) Comparison between the absorption spectra of the absorbers with the PSO and corresponding rounded values. (**f**) Spectral response of the absorber with the rounded values. The figure illustrates the resonance wavelengths along with their associated absorption values.

The absorption behavior of an absorber strongly depends on its constituent materials, geometric shape, and dimensions. The constituent materials are important because each material has a unique wavelength-dependent permittivity spectrum. This variability causes the absorber’s behavior to differ at different wavelengths. Moreover, the geometric shape and dimensions of the absorber are crucial for exciting various resonances, such as SPPs and LSPRs. By selecting the appropriate materials and designing resonators with optimal dimensions, it is possible to create an absorber that maximally captures incident light. Optimization algorithms offer one the best approaches to obtain the optimal geometric variable values. The optimization of the absorber geometry was performed using the PSO algorithm, which iteratively updates a population of candidate solutions (“particles”) by balancing exploration and exploitation of the search space^46^. Each particle adjusts its position based on its own best historical solution and the global best solution found by the swarm. The velocity update follows:

3 $$v_{i}^{{t+1}}=wv_{i}^{t}+{c_1}{r_1}({p_i} - x_{i}^{t})+{c_2}{r_2}(g - x_{i}^{t})$$

where *w* is the inertia weight, *c*_1_ and *c*_2_ are the cognitive and social coefficients, *r*_1_ and *r*_2_ are random numbers uniformly distributed in [0,1], and *x*_*i*_^*t*^ is the position vector at iteration *t*^47^. In our simulations, the swarm consisted of 10 particles and was iterated 50 times (500 evaluations in total). Convergence was defined as the iteration at which the improvement of the global best figure of merit (FOM) (average absorption over 0.25–4 μm) was less than 0.01% for five successive iterations. These settings yielded reproducible results across three independent runs, confirming the robustness of the optimization procedure. The corresponding flowchart of the used PSO algorithm is shown in Fig. 3(a). The algorithm begins with randomly chosen geometric values and then calculates the FOM. Two parameters, namely maximum generation number and generation size, dictate the number of simulations. The total number of simulations is determined by multiplying the generation size by the maximum number of generations. The highest FOM is chosen as the local best during each generation. If the new local best exceeds the highest FOM recorded historically (global best), it becomes the new global best. This process is repeated until the maximum generation size is attained^48^.

To simplify the manufacturing process, the dimensions of each resonator are designed to be smaller than or equal to those of the resonator beneath it. As a result, each resonator is fully positioned on the surface of its underlying resonator. To establish constraints on the dimensions of the resonators, three geometric coefficients are used as guidelines: *k*_1_ = *d*/*w*_1_, *k*_2_ = *w*_2_/*d*, and *k*_3_ = *w*_3_/*w*_2_. By using these geometric coefficients and setting a maximum limit of “1” for *k*_1_ and *k*_3_, along with “0.706” for *k*_2_, we ensure that the dimensions of each resonator do not exceed the dimensions of the resonator below it. Therefore, the variables of the PSO algorithm are *h*_1_, *h*_2_, *h*_3_, *h*_4_, *h*_5_, *w*_1_, *k*_1_, *k*_2_, and *k*_3_. Table 1 lists the range of variations of each variable in the PSO algorithm.

**Table 1:** The variation range of each variable in the PSO algorithm.

Variable	Lowest value	Highest value
*h*_1_ (nm)	20	250
*h*_2_ (nm)	20	250
*h*_3_ (nm)	20	250
*h*_4_ (nm)	20	250
*h*_5_ (nm)	20	250
*w*_1_ (nm)	200	400
*k*_1_ = *d*/*w*_1_	0.5 (*d* = 100 nm)	1 (*d* = 400 nm)
*k*_2_ = *w*_2_/*d*	0.5 (*w*_2_ = 50 nm)	0.706 (*w*_2_ = 282 nm)
*k*_3_ = *w*_3_/*w*_2_	0.5 (*w*_3_ = 25 nm)	1 (*w*_3_ = 282 nm)

To design a broadband absorber that incorporates the sunlight spectrum, the FOM of the algorithm is defined in the form of average absorption, denoted as A_avg_ with λ_min_ = 0.25 μm and λ_max_ = 4 μm.

4 $${A_{avg}}=\frac{1}{{{\lambda _{\hbox{max} }} - {\lambda _{\hbox{min} }}}}\int_{{{\lambda _{\hbox{min} }}}}^{{{\lambda _{\hbox{max} }}}} {A\left( \lambda \right)d\lambda }$$

Figures 3(b) and (c) illustrate the geometric variables (*h*_1_, *h*_2_, *h*_3_, *h*_4_, *h*_5_, and *w*_1_) and the geometric coefficients (*k*_1_, *k*_2_, and *k*_3_) as a function of the generation number. The values for the FOM at each generation number are presented in Fig. 3(d). At the generation number equal to 1, the FOM value is 0.9495. As the generations progress, the FOM value improves. Ultimately, the proposed absorber achieves its peak FOM value of 0.9902 at a generation number of 29. This value is the best FOM for the proposed absorber because the FOM does not change for generation numbers beyond 29. The values generated by the PSO algorithm have several decimal places below the nanometer scale. Therefore, we have rounded these numbers to the suitable integer. Table 2 displays the values obtained from the PSO algorithm and the corresponding rounded values. Figure 3(e) illustrates the impact of rounding the values obtained from the PSO algorithm on the absorber’s absorption spectrum. This rounding leads to a reduction in A_avg_ by 0.08%, decreasing it from 0.9902 to 0.9894. In the remainder of the paper, the rounded values ​​are considered as the original values ​​of the geometric variables of the absorber. The absorption, reflection, and transmission spectra of the optimized absorber, with a Ti substrate thickness of 150 nm and a periodicity of *P* = 400 nm, are presented in Fig. 3(f). The over 90% absorption bandwidth spans 3533 nm, ranging from 467 nm to 4000 nm. Notably, five resonances appear in the absorption spectrum, which are shown in the figure. Moreover, absorption exceeds 99% in four distinct bands of 555–1197 nm, 1515–2109 nm, 2483–3058 nm, and 3406–3963 nm.

**Table 2:** Values obtained from the PSO algorithm and the corresponding rounded values.

Variables	PSO values	Rounded values
*h*_1_ (nm)	41.01	40
*h*_2_ (nm)	250	250
*h*_3_ (nm)	22.35	20
*h*_4_ (nm)	250	250
*h*_5_ (nm)	86.53	85
*w*_1_ (nm)	297.6	300
*k*_1_ = *d*/*w*_1_	1 (*d* = 297.6 nm)	1 (*d* = 300 nm)
*k*_2_ = *w*_2_/*d*	0.6223 (*w*_2_ = 185.2 nm)	0.623 (*w*_2_ = 187 nm)
*k*_3_ = *w*_3_/*w*_2_	1 (*w*_3_ = 185.2 nm)	1 (*w*_3_ = 187 nm)

**Fig. 4:**
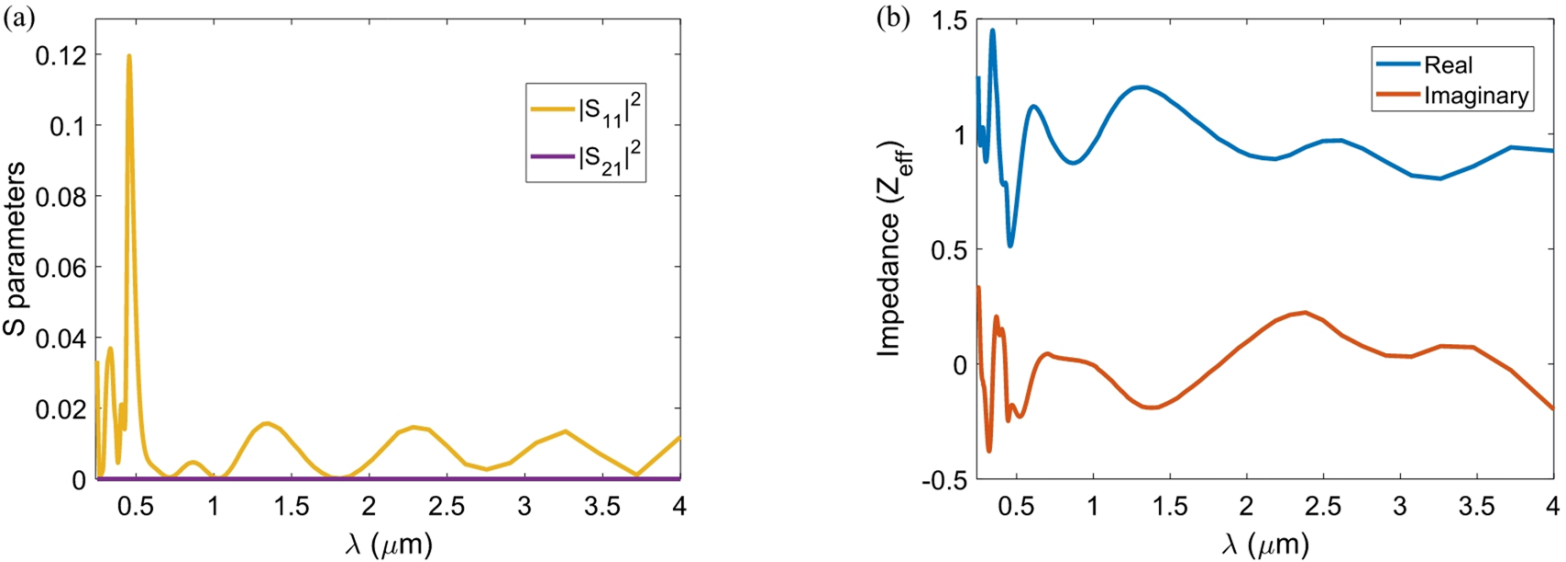
(**a**) Simulated reflection (|S_11_|^2^) and transmission (|S_21_|^2^) spectra of the optimized Ti/TiO_2_ absorber. (**b**) Normalized effective impedance (*Z*_*eff*_) spectrum of the absorber, illustrating the real part of the impedance, which is close to unity, and the near-zero imaginary part across the 0.25–4.25 µm range, confirming strong impedance matching with free space.”.

The impedance matching between the structure and free space influences the reflection value. When the conditions for impedance matching are met, incident light penetrates the absorber and is absorbed by the lossy dielectric material. To achieve maximum absorption, the normalized effective impedance of the structure (*Z*_*eff*_) must match the impedance of free space (*Z*_0_). The *Z*_*eff*_ can be expressed in terms of the S-parameters of the scattering matrix, as follows^49^:

5 $${Z_{eff}}=\sqrt {\frac{{{{\left( {1+{S_{11}}} \right)}^2} - {S_{21}}^{2}}}{{{{\left( {1 - {S_{11}}} \right)}^2} - {S_{21}}^{2}}}}$$

where |*S*_11_|^2^ and |*S*_21_|^2^ indicate the reflection and transmission parameters, respectively^50^. The |*S*_11_|^2^ and |*S*_21_|^2^ spectra as well as the real and imaginary parts of the *Z*_*eff*_ are illustrated in Figs. 4(a) and (b), respectively. The figure exhibits a real part close to unity and a near-zero imaginary part across the 0.25–4 μm range, confirming strong impedance matching with free space. Figure 4 validates the spectral response of the absorber shown in Fig. 3(f).

Multiple resonance mechanisms, including SPPs, MPs, LSPRs, and CRs, influence the resonant characteristics of the proposed absorber. These mechanisms are significantly affected by the arrangement, materials, and dimensions of the absorber layers. SPPs are collective electromagnetic waves at the interface between a metal and a dielectric^51^. MPs can be excited by alternating layers of metal and dielectric^52^. LSPRs are non-propagating SPPs confined to nanostructured surfaces^53^. Additionally, a quasi-cavity is formed by placing four unit cells adjacent to one another, leading to CRs. The SPP resonance wavelength, denoted as λ_SPP_, at a metal-dielectric interface (specifically Ti/TiO_2_) is expressed by the equation λ_SPP_ = λ_0_[(*ε*_*m*_ + *ε*_*d*_)/(*ε*_*m*_ *ε*_*d*_)]^1/2^. In this equation, λ_0_ represents the incident light wavelength, while *ε*_*m*_ and *ε*_*d*_ denote the permittivities of Ti and TiO_2_, respectively^54,55^. For the Ti/TiO_2_ disk or square resonators, the LSPR resonant wavelength can be calculated using the formula λ_LSPR_ = 2π*c*/*ω*_LSPR_, where *ω*_LSPR_ = *ω*_p_/(1 + 2*ε*_*d*_)^1/2^. In this context, *ω*_*p*_ represents the plasma frequency of Ti, and *c* is the speed of light^56,57^. The resonance wavelength λ_MP_ for MP modes in metamaterial structures can be described using an LC resonance model represented by λ_MP_ ≈ 2π*c*[(*L* + *M*)*C*]^1/2^. Here, *L* denotes the effective inductance (considering geometric contributions), *M* stands for mutual inductance, and *C* is the effective capacitance, which is influenced by the geometry and separation of the Ti and TiO_2_ layers^58,59^. For a meta-cavity bounded by a resonant metasurface and a metallic reflector (Ti film), a Fabry-Perot-like cavity is formed with a resonance wavelength of λ_cav_ = 2*n*_*eff*_*T*_*eff*_/*m*, where *n*_*eff*_ is the effective refractive index (averaged across Ti/TiO_2_ layers), *T*_*eff*_ is the total optical cavity thickness (sum of Ti and TiO_2_ film thicknesses), and *m* denotes the resonance mode number (typically *m* = 1 for the fundamental mode)^60^. Additionally, another quasi-cavity is formed by arranging four unit cells adjacent to one another, leading to the periodicity of coupled resonators.

The simultaneous excitation and mutual coupling of these distinct resonance modes collectively contribute to the absorber’s broadband performance. Specifically, the hybridization between SPPs and LSPRs enhances optical confinement at short wavelengths, while the interaction of MPs and CRs broadens the absorption toward the infrared region. This mode hybridization creates multiple overlapping absorption peaks, effectively merging them into a continuous wideband response. Such synergistic coupling between electric and magnetic resonances improves impedance matching across a broad spectral range, thereby minimizing reflection and ensuring near-unity absorption throughout the 0.25–4 μm region.

**Fig. 5:**
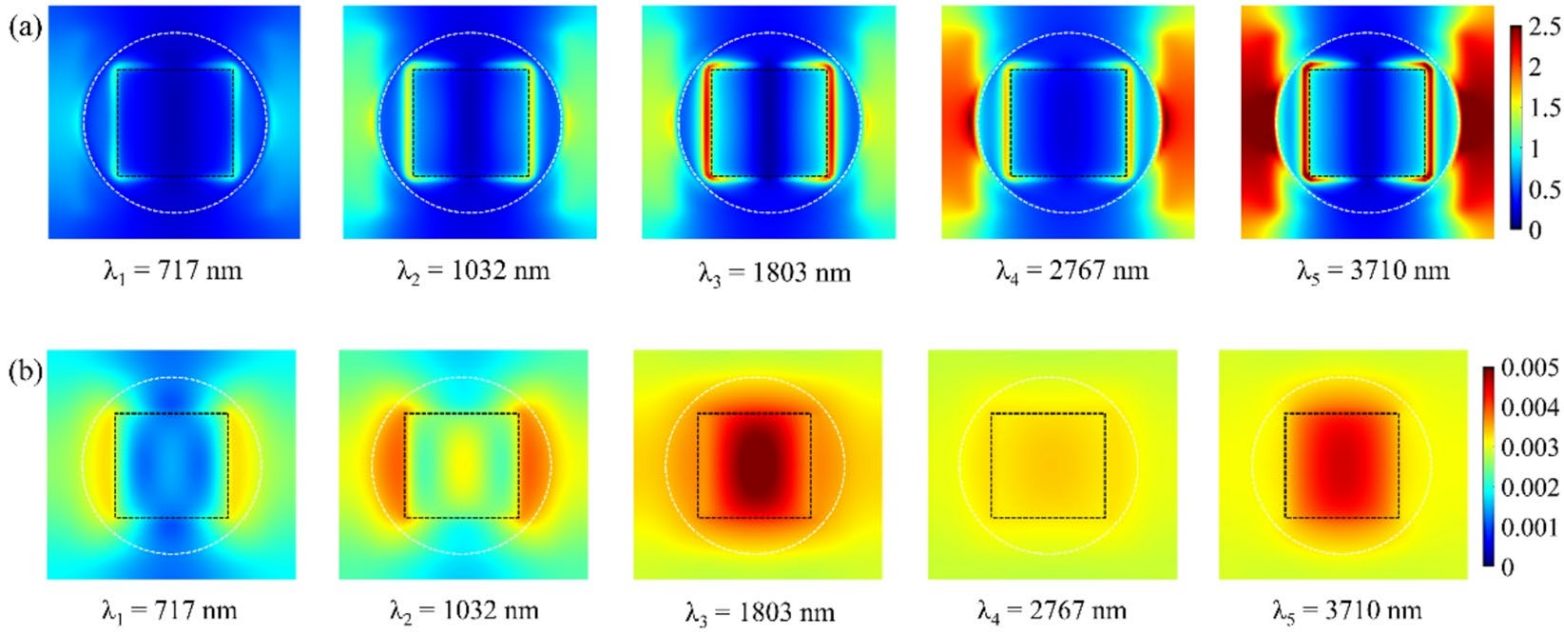
Spatial distribution of the electric (|*E*|) and magnetic (|*H*|) field intensities in the *x*-*y* plane at five resonance wavelengths. The |*E*| fields exhibit strong localization at the edges of Ti/TiO_2_ resonators and inter-cell gaps, indicative of LSPRs and CRs. The |*H*| field maps confirm the MP contributions, particularly at longer wavelengths.

**Fig. 6:**
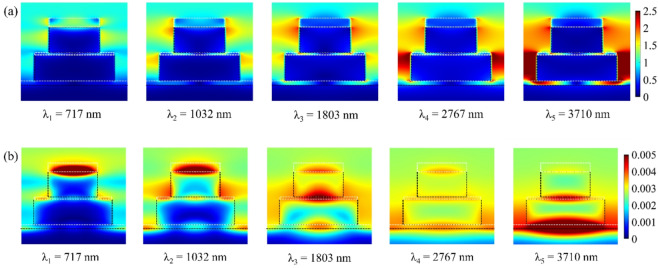
Spatial distribution of the electric (|*E*|) and magnetic (|*H*|) field intensities in the *x*-*z* plane at five resonance wavelengths. The |*E*| fields are predominantly confined to the upper resonator interfaces, highlighting vertical plasmonic interactions. The |*H*| fields show mode confinement in TiO_2_ layers, validating the excitation of magnetic polaritons. These field profiles together illustrate how hybrid resonance mechanisms enable ultra-broadband absorption.

The electric (|*E*|) and magnetic (|*H*|) field magnitude distributions at resonance wavelengths can be analyzed using surface monitors positioned in the *x*-*y* and *x*-*z* planes. This configuration facilitates the evaluation of how each resonance mode influences the absorption spectrum. Figure 5 depicts the |*E*| and |*H*| field distributions in the *x*-*y* plane, where monitors placed at the interface between the TiO_2_ disk resonator and the upper Ti square resonator reveal field patterns at resonance wavelengths λ_1_ = 717 nm, λ_2_ = 1032 nm, λ_3_ = 1803 nm, λ_4_ = 2767 nm, and λ_5_ = 3710 nm. Meanwhile, Fig. 6 presents the field distributions in the *x*-*z* plane. The results from Figs. 5 and 6 demonstrate that the electric field is predominantly localized along the resonator edges and in the gaps between adjacent unit cells (on the outer regions of the resonators). The high electric field intensity in the intercellular gaps suggests robust coupling between the resonators of neighboring cells, attributed to CRs. Additionally, the enhanced electric field at the resonator edges arises from LSPRs.

In contrast, the magnetic field distributions exhibit distinct behavior. At shorter wavelengths, the magnetic field is mainly confined within the TiO_2_ square resonator due to SPP excitation. However, as the wavelength increases, the magnetic field shifts toward the underlying TiO_2_ dielectric layers situated between the Ti metal layers, a consequence of MP excitation. A comparison of resonance mechanisms indicates that SPPs and LSPRs predominantly enhance absorption at shorter wavelengths, whereas CRs and MPs play a more significant role at longer wavelengths. By combining these resonant effects, a broad and efficient absorption spectrum can be achieved. Table 3 summarizes the modal decomposition based on field confinement regions, spatial overlap, and resonator interactions visible in the simulation results.

**Table 3:** Contributions of resonator interactions in the absorption spectrum of the absorber.

Resonance wavelength	Dominant |E| field location	Dominant |H| field location	Primary resonance mechanisms
λ_1_ = 717 nm	Edges of the Ti square/disk resonators; intercell gaps	Near Ti/TiO_2_ interface; TiO_2_ square layer	LSPR + SPP
λ_2_ = 1032 nm	Ti square corners and inter-resonator gaps	Localized in TiO_2_ near the upper resonators	LSPR + SPP
λ_3_ = 1803 nm	Broader |*E*| localization across disk and square	Deep field penetration in the TiO_2_ layers	MP + weak LSPR
λ_4_ = 2767 nm	Intercell gaps and between the stacked layers	Confined between the lower Ti and TiO_2_ layers	MP + CR
λ_5_ = 3710 nm	Field concentration in the deep multilayer cavities	Strong MP localization in the lower dielectric	MP + CR

**Fig. 7:**
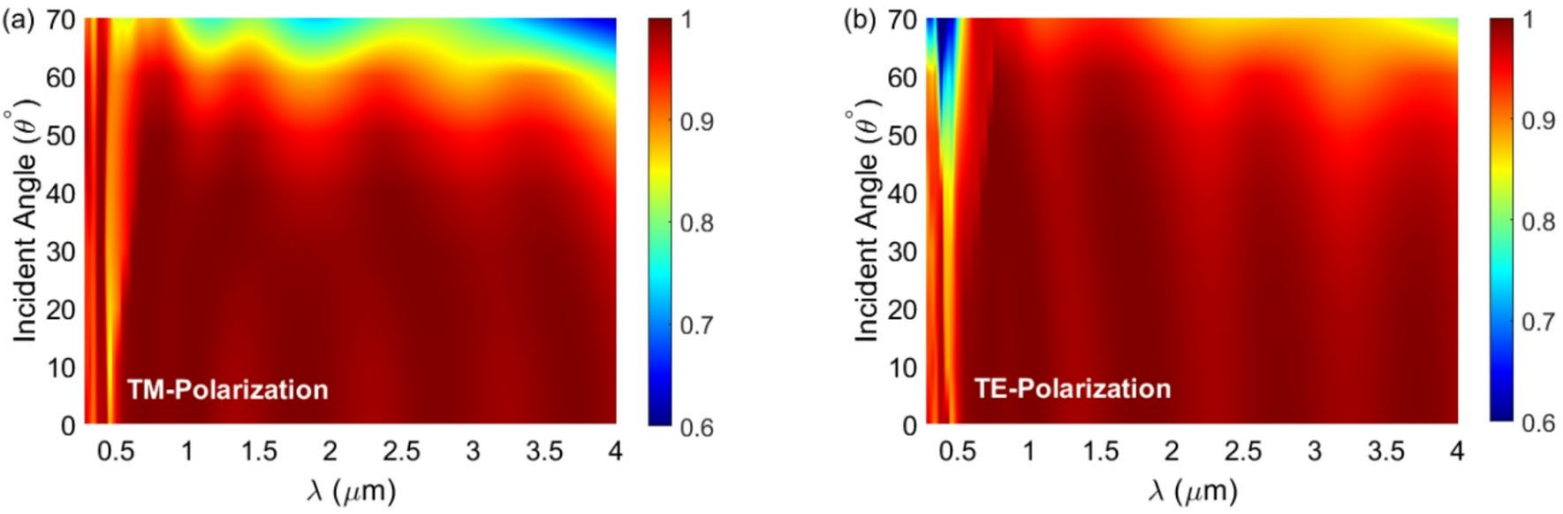
Absorption spectrum of the optimized absorber when subjected to oblique incidence angles varying from 0° to 70° for (**a**) TM polarization and (**b**) TE polarization.

In addition to other factors, the absorption behavior of an absorber depends on the angle and polarization of the incident light. When the incident wave strikes the surface of the absorber perpendicularly, the absorber operates independently of the polarization of the incoming wave due to its symmetrical properties. However, the response of the absorber to transverse magnetic (TM) and transverse electric (TE) polarizations differs for oblique radiation. Figure 7 illustrates the absorption characteristics of the optimized absorber for both TM and TE polarizations at various incident angles, ranging from *θ* = 0° to *θ* = 70°. For TM polarization, the absorption remains above 80% even at an incident angle of 60° across the entire wavelength range examined. Specifically, the absorption drops to 81.25% at λ = 4 μm for an incident angle of *θ* = 60°. In contrast, the absorption spectrum for TE polarization reveals different behavior, with absorption decreasing to 61.44% at *θ* = 60° and λ = 385 nm. Ignoring short wavelengths up to approximately 0.6 μm, the absorber exhibits more than 90% absorption for light with TE polarization at an incident angle of 60°. By comparing the data shown in Fig. 7, we can conclude that up to an incident angle of 50°, the absorber functions independently of both polarization and the angle of the incoming light. As the incident angle increases, the absorber performs better at short wavelengths for TM polarization and at long wavelengths for TE polarization. Additional details are provided in the Supplementary Material.

As previously discussed, the wavelength range of the absorber’s operation has been chosen to cover the sunlight spectrum completely. In this case, the proposed absorber is also well suited for solar applications. For this purpose, the solar absorption (*η*_A_) of the absorber, which presents optical-to-thermal efficiency under AM1.5G illumination, is calculated as follows^61^:

6 $${\eta _{\mathrm{A}}}=\frac{{\int_{{{\lambda _{\hbox{min} }}}}^{{{\lambda _{\hbox{max} }}}} {A\left( \lambda \right){I_{{\mathrm{AM1}}{\mathrm{.5G}}}}\left( \lambda \right)d\lambda } }}{{\int_{{{\lambda _{\hbox{min} }}}}^{{{\lambda _{\hbox{max} }}}} {{I_{\mathrm{A}}}_{{{\mathrm{M1}}{\mathrm{.5G}}}}\left( \lambda \right)d\lambda } }}$$

**Fig. 8:**
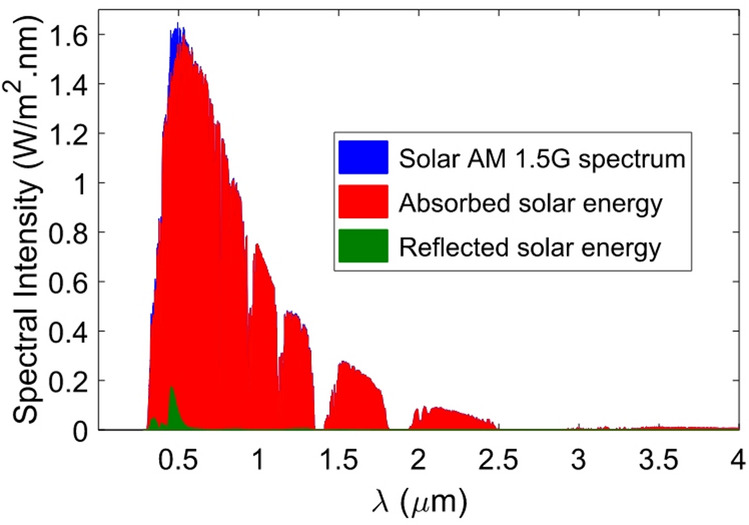
Spectral absorption and reflection profiles of the designed absorber under AM 1.5G solar irradiation.

In this context, the incident AM 1.5G solar power spectrum is represented by *I*_AM1.5G_(λ). For the given minimum wavelength of λ_min_ = 0.28 μm and maximum wavelength of λ_max_ = 4 μm, the *η*_*A*_ is calculated to be 0.9817. In other words, the designed absorber exhibits exceptional efficiency, capturing 98.17% of incident solar radiation across the 0.28–4 μm spectral range. Figure 8 illustrates the AM 1.5G solar spectrum with the absorbed and unharvested energy components. Using the simulated absorption A(λ) and the AM1.5G spectrum *I*_AM1.5G_(λ), the absorbed power per unit area can be calculated by:

7 $$\frac{{{P_{{\mathrm{abs}}}}}}{{{A_{{\mathrm{device}}}}}}=\int_{{{\lambda _{\hbox{min} }}}}^{{{\lambda _{\hbox{max} }}}} {A\left( \lambda \right){I_{{\mathrm{AM1}}{\mathrm{.5G}}}}\left( \lambda \right)d\lambda }$$

The total absorbed power is represented as P_abs_ (W), and the device area is denoted as A_device_ (m^2^). When an absorber is placed on a thermally conductive substrate, such as silicon (Si) or copper (Cu), which is connected to a thermoelectric module, the local temperature rise (ΔT ≈ P_abs_/G_th_) caused by the absorbed light generates a Seebeck voltage (V_S_ = *S*ΔT) across the thermoelectric junctions^62^. In a thermophotovoltaic (TPV) configuration, the absorber can also function as a selective emitter, converting the absorbed broadband sunlight into re-emitted infrared radiation that matches the bandgap of a TPV cell^63^. In this context, G_th_ refers to the thermal conductance of the substrate and thermal contacts, while *S* represents the Seebeck coefficient (V.K^−1^) of the thermoelectric material or module.

**Fig. 9:**
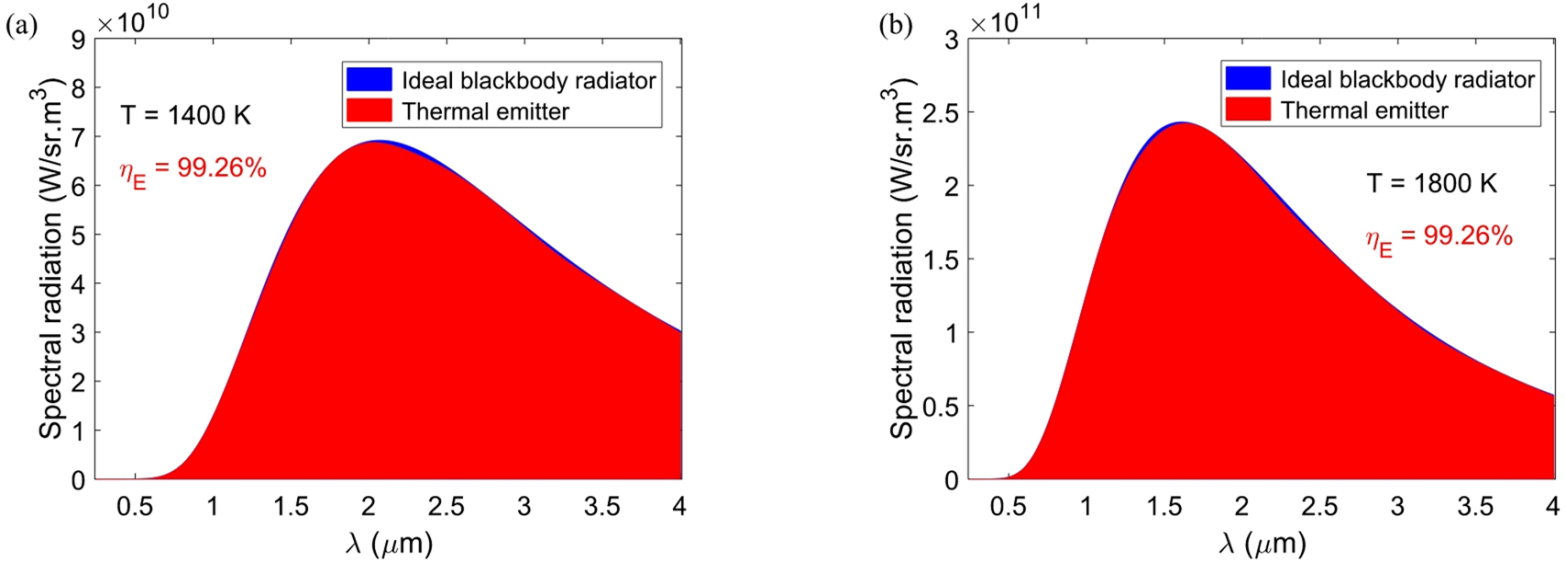
Thermal emissivity of the structure under temperatures of (**a**) 1400 K and (**b**) 1800 K. This figure shows that the thermal emission efficiency exceeds 99% across a wide range of temperatures.

As expressed, an absorber can also be considered as a highly efficient thermal emitter. Any material with a temperature above absolute zero radiates thermal energy. According to Kirchhoff’s law, the thermal emittance of a material is equal to its absorption. To assess the effectiveness of the thermal emitter, a key metric known as thermal emission efficiency (*η*_*E*_) is defined as follows^64^:

8 $${\eta _E}=\frac{{\int_{{{\lambda _{\hbox{min} }}}}^{{{\lambda _{\hbox{max} }}}} {A\left( \lambda \right){I_{BE}}\left( {\lambda ,T} \right)d\lambda } }}{{\int_{{{\lambda _{\hbox{min} }}}}^{{{\lambda _{\hbox{max} }}}} {{I_{BE}}\left( {\lambda ,T} \right)d\lambda } }}$$

where *I*_*BE*_(λ,*T*) denotes the ideal blackbody spectral intensity at wavelength λ and temperature *T*. Figure 9 illustrates the thermal emissivity of the absorber at *T* = 1400 K and *T* = 1800 K. At both temperatures, the absorber demonstrates an exceptional *η*_*E*_ of 99.26%. Consequently, this emitter lays the groundwork for developing ideal and high-efficient thermal emitters, suitable for high-temperature applications. It is important to note that we have not considered the temperature-dependent permittivity of Ti and TiO_2_. The permittivity (both real and imaginary components) of materials can vary significantly with an increase in temperature, impacting resonant modes and, consequently, altering absorption and emission performance.

**Fig. 10:**
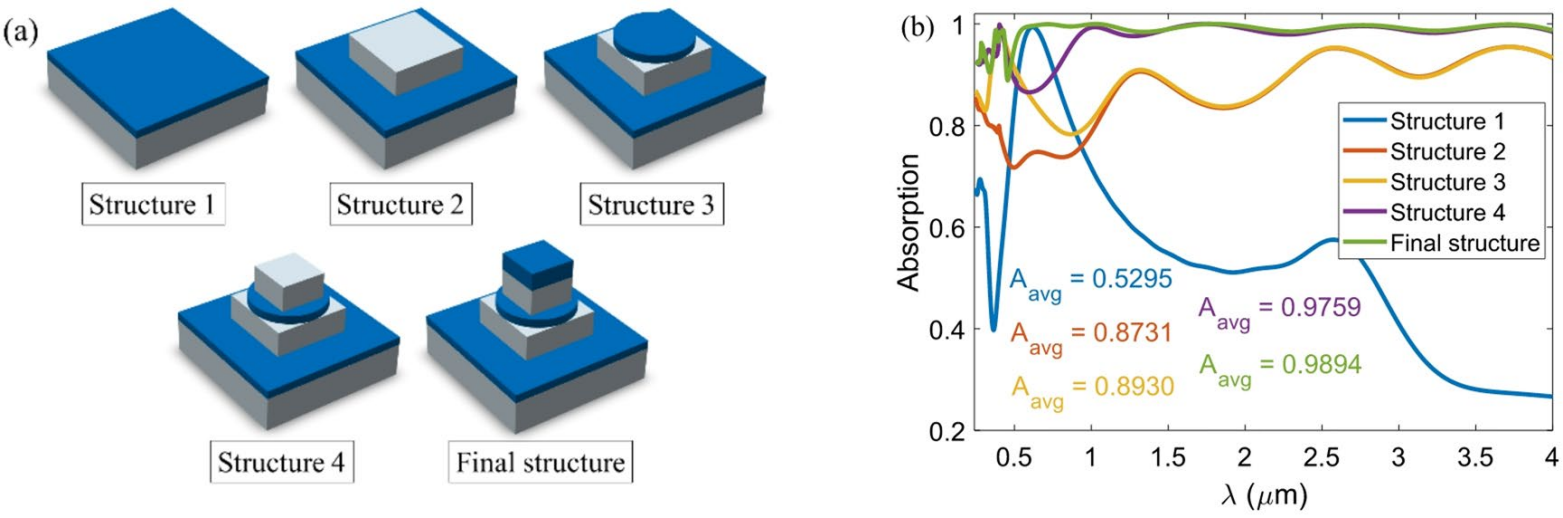
(**a**) Different states of the proposed absorber structure and (**b**) absorption spectrum for each structure.

Further exploration focuses on the impact of the presence of each resonator on the absorption spectrum of the absorber. Figure 10 compares the absorption performance of the absorber across different structural configurations, highlighting the individual and collective contributions of each resonator layer. In “Structure 1”, which contains only Ti and TiO_2_ thin films, the absorption is relatively low (A_avg_ = 52.95%). This amount of absorption is also due to the excitation of SPPs between the TiO_2_ dielectric layers and the Ti metal. Adding a Ti square resonator in “Structure 2” significantly enhances absorption (A_avg_ = 87.31%). This improvement is achieved by facilitating SPPs, CRs, and LSPRs, which concentrate the electric field at the metal-dielectric interface, periodic cavities created, and corners of the resonators. The square resonator supports absorption in short and long wavelength regions by resonantly exciting these modes. “Structure 3” introduces a TiO_2_ disk resonator, further improving absorption within the 0.25–1.314 μm range with an A_avg_ of 89.3%. This structure introduces additional LSPR and SPP modes. These resonances promote broader field confinement and enhance light-matter interactions, particularly in the visible and near-infrared regions. In “Structures 4” and “Final structure”, additional Ti and TiO_2_ square resonators increase the resonant layers. These configurations stimulate MPs due to the alternating dielectric-metal layers and lead to improved impedance matching with free space. As a result, these structures support multi-band resonances and broader spectral coverage, achieving ultra-broadband absorption with A_avg_ values of 97.59% and 98.94%, respectively. These findings confirm that each additional geometric feature contributes to the synergistic excitation of hybrid resonant modes (SPPs, LSPRs, MPs, and CRs), which enhance both the spectral breadth and intensity of absorption together.

The proposed Ti/TiO_2_ multilayer absorber can be fabricated using standard atomic layer deposition (ALD) for the dielectric layers, along with electron beam lithography (EBL) or nanoimprint lithography (NIL) for patterning the metallic resonators. ALD provides sub-nanometer thickness control and ensures uniform, conformal coating even on high-aspect-ratio structures, which is crucial for maintaining structural accuracy at the nanoscale^65^. For patterning Ti, EBL offers precise lateral resolution of less than 10 nm, while NIL allows for scalable replication at a significantly lower cost^66^. To mitigate interfacial stress arising from the thermal expansion mismatch between Ti (α ≈ 8.6 × 10^− 6^ K^−1^) and TiO_2_ (α ≈ 9 × 10^− 6^ K^−1^), intermediate annealing steps (e.g., 400 °C in N_2_) can relieve built-in strain and enhance adhesion^67^. Additionally, introducing a thin titanium nitride (TiN) adhesion or diffusion barrier layer can improve thermal stability for operations at elevated temperatures. In terms of cost-effectiveness and scalability, replacing EBL with NIL or photolithography for large-area patterning, and using magnetron sputtering instead of physical vapor deposition (PVD) for Ti films, can significantly reduce production costs while preserving optical performance. Moreover, numerical studies on material substitution were conducted by keeping the geometry consistent while varying the metallic and dielectric materials. It was found that alternatives such as TiN, tungsten (W), and chromium (Cr) as refractory metals, and aluminum oxide (Al_2_O_3_) or silicon nitride (Si_3_N_4_) as dielectric spacers, can maintain more than 90% absorption across comparable bandwidths, despite slight shifts in the exact spectral positions. These findings indicate that the proposed design concept is flexible regarding materials and can be adapted by adjusting the geometrical parameters for various applications, including high-temperature emitters and cost-sensitive solar absorbers. Current nanofabrication methods can reliably achieve the precision required for the proposed Ti/TiO_2_ structure. Electron-beam lithography offers overlay accuracies of tens of nanometers, while nanoimprint lithography with moiré-fringe alignment achieves sub-20 nm overlay, and atomic layer deposition ensures angstrom-level thickness control, enabling accurate multilayer registration and conformal interfaces^68^.

**Fig. 11:**
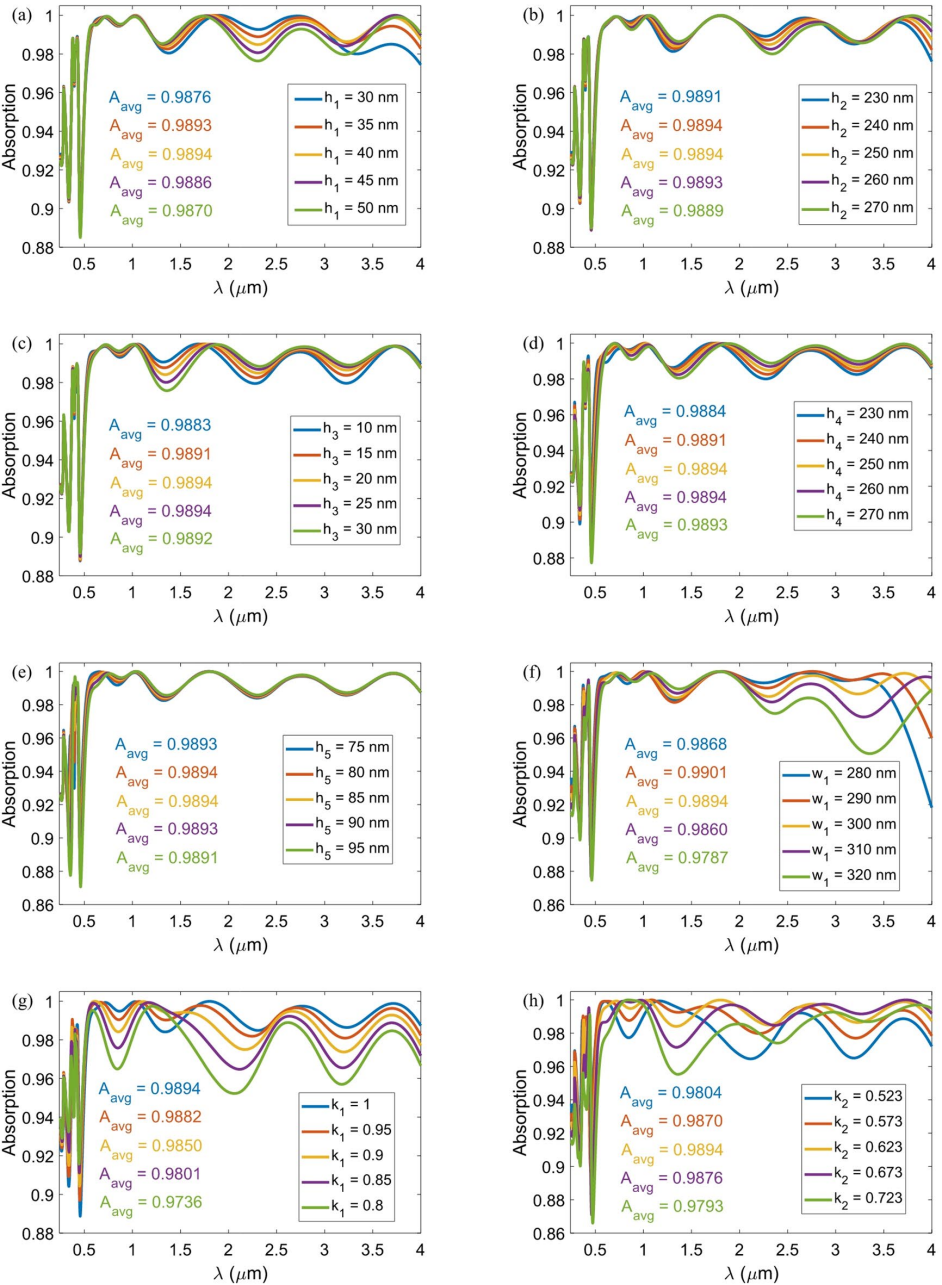
Absorption spectra of the absorber for relatively minor changes in the geometric variables of (**a**) *h*_1_, (**b**) *h*_2_, (**c**) *h*_3_, (**d**) *h*_4_, (**e**) *h*_5_, (**f**) *w*_1_, (**g**) *k*_1_, (**h**) *k*_2_, (**i**) *k*_3_, and (**j**) *P*.

**Fig. 12:**
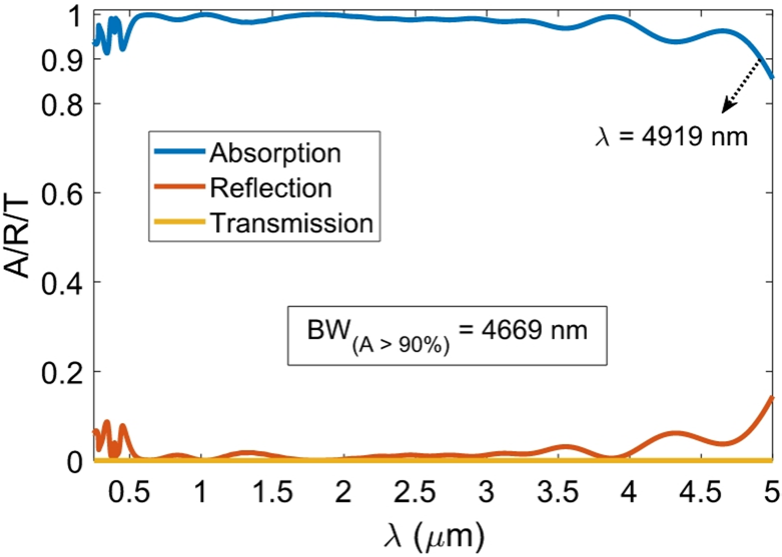
Spectral response of the absorber with rounded optimal values and *k*_3_ = 0.9 within an extended wavelength range up to 5 μm.

Errors in the fabrication process are inevitable and can impact the performance of any device. For this reason, we have investigated the absorption behavior of the proposed absorber in response to relatively small changes in the geometric variables and the structural period. A close examination of the A_avg_ values listed in Fig. 11 reveals that the proposed absorber is highly robust to the probabilistic errors of the fabrication process, with the A_avg_ value does not drop below 0.9736. In addition, when the period is changed to 360 nm, the A_avg_ value is still a commendable 0.9677. Additionally, when we adjust *w*_1_ from 300 nm to 290 nm, keeping all other parameters constant, the A_avg_ increases to 0.9901, close to the optimal 0.9902 achieved through the PSO algorithm. It is important to note that the absorption value of the absorber with rounded geometric dimensions is below 90% in the wavelength range of 449 to 467 nm. However, by adjusting the geometric coefficient of *k*_3_ to 0.9, we can increase the A_avg_ to 0.9899. This adjustment enables us to achieve an absorption spectrum that exceeds 92% across the wavelength range of 0.25 to 4 μm. As a result, the over 90% absorption bandwidth of the absorber is the entire studied wavelength range, which is 3750 nm. Since the absorption in the relevant range exceeds 90%, it is advisable to expand the wavelength range of the simulation to determine the cutoff wavelength for 90% absorption of the absorber. Therefore, the wavelength range of the simulation has been extended to 5 μm. Figure 12 demonstrates that the absorber effectively absorbs 90% of the incident light within the wavelength range of 250 to 4919 nm, resulting in an absorption bandwidth of 4669 nm for the absorber.

Table 4 compares the constituent materials, over 90% absorption bandwidth, and bandwidth range of the presented absorber with that of previously reported Ti-based absorbers. The absorber analyzed in this work exhibits the widest reported > 90% absorption bandwidth among Ti/TiO_2_-based metamaterial absorbers to date, spanning 4669 nm (250–4919 nm), as benchmarked in Table 4. In this table, the works have been sorted by bandwidth value.

**Table 4:** Comparison between the characteristics of the proposed absorber and previously reported Ti-based absorbers.

References	Material		A > 90% bandwidth	Bandwidth range	Unit cell configuration
	Metal	Dielectric			
^25^	Ti	SiO_2_	1555 nm	421–1976 nm	Ti disk resonators on SiO_2_-Ti layers
^26^	Ti	SiO_2_	1576 nm	329–1905 nm	Ti-SiO_2_-Si square on SiO_2_-Ti layers
^27^	Ti	SiO_2_	1650 nm	550–2200 nm	SiO_2_ anti-reflection layer on Ti resonators, all located on SiO_2_-Ti-Si layers
^7^	Ti	SiO_2_	1700 nm	300–2100 nm	SiO2-Ti square bilayer grating arrays on SiO_2_-Ti layers
^69^	Ti	SiO_2_	2100 nm	400–2500 nm	Cross-shaped Ti resonators with cylinder-shaped central resonant cavities on SiO_2_-Ti layers
^29^	Ti	TiO_2_	3000 nm	200–3200 nm	Ti-TiO_2_-Ti square disk resonators, surrounded by a Ti square ring resonator, all located on TiO_2_-Ti layers
^70^	Ti	TiO_2_	3509 nm	392–3901 nm	Ti disk resonator on a stack of TiO_2_-Ti square resonators, all placed on TiO_2_-Ti layers
This work	Ti	TiO_2_	4669 nm	250–4919 nm	TiO_2_-Ti square resonators along with a TiO_2_ disk resonator and a Ti square resonator, all positioned on TiO_2_-Ti layers

Looking ahead, there are several ways to further enhance research on broadband metamaterial absorbers. The incorporation of innovative materials, such as refractory ceramics (e.g., TiN, HfN) or phase-stable oxides (e.g., Al_2_O_3_, Si_3_N_4_), is likely to improve thermal stability, expand operational bandwidth, and enable multifunctional capabilities, including sensing and emission control^71^. Furthermore, the integration of intelligent or active control methods, such as phase-change materials (e.g., VO_2_, GST) and techniques for electro-optic or microfluidic tuning, holds promise for achieving dynamically reconfigurable absorption and emission spectra^72^. Employing machine-learning-based inverse design approaches may accelerate the optimization of geometries and facilitate real-time spectral adjustments^73^. In addition, applying these advanced materials in practical settings, such as solar thermal collectors, thermophotovoltaic systems, and infrared stealth coatings, will provide critical validation and refinement of theoretical models in real-world operational environments. The experimental implementation of scalable fabrication techniques, such as nanoimprint lithography and sputtering, will serve to bridge the gap between simulation results and device performance. These strategies not only enhance our understanding of light-matter interactions within metamaterials but also expand their potential applications in energy conversion and photonic regulation.

## Conclusion

This study successfully designed and optimized an ultra-broadband metamaterial absorber using Ti and TiO_2_ resonators, achieving an average absorption of 98.99% across 0.25–4 μm. The absorber with rounded geometric values and *k*_3_ = 0.9 captures over 90% of the incoming light within the wavelength range of 250–4919 nm, which indicates a bandwidth of 4669 nm. The absorber demonstrated excellent polarization independence (up to 50°) and angular stability, maintaining over 80% absorption even at a 60° incidence. Additionally, it achieved a solar absorption efficiency of 98.17% under AM 1.5G illumination and thermal emission efficiency of 99.26% at a temperature of 1800 K. Field analysis revealed that the combined effects of SPPs, MPs, LSPRs, and CRs contributed to its broadband performance. Fabrication tolerance studies confirmed its robustness against geometric variations. With its ultra-broadband response and near-perfect absorption, this design offers significant potential for solar energy harvesting and other optoelectronic applications.

## Supplementary Information

Below is the link to the electronic supplementary material.

Supplementary Material 1

## Data Availability

The datasets used and/or analyzed during the current study are available from the corresponding author on reasonable request.
