# Supplementary material for: Design and optimization of a polarization-insensitive Ti/TiO2 metamaterial absorber using particle swarm optimization for broadband solar–thermal applications
Source: Sci Rep. 2025 Dec 15;16:2089. doi: 10.1038/s41598-025-31966-3 (PMC12808734; doi:10.1038/s41598-025-31966-3)
Supplement: Supplementary file 1 — Supplementary Material 1 [file 41598_2025_31966_MOESM1_ESM.docx]

**Supplementary Material**

**S1. Simulation Details**

To reproduce the numerical results presented in this work, the following simulation settings and parameters were employed:

**Software:**

- *Ansys Lumerical FDTD*, Version 2020 R2.4

**Materials:**

- **Metal:** Titanium (Ti); optical constants obtained using the CRC model available in the Lumerical material database.
- **Dielectric:** Titanium dioxide (TiO_2_); refractive index data imported according to Siefke *et al.* ^1,2^.

**Excitation Source:**

- Plane-wave source of Bloch/periodic type
- Spectral bandwidth: 0.25–4 µm

**Boundary Conditions:**

- ***x*-direction:** Anti-Symmetric
- ***y*-direction:** Symmetric
- ***z*-direction:** Perfectly Matched Layer (PML)

**Mesh Settings:**

- Uniform mesh size of 5 nm in all spatial directions

**Optimal Geometrical Parameters:**

- *h*_1_ = 40 (nm)
- *h*_2_ = 250 (nm)
- *h*_3_ = 20 (nm)
- *h*_4_ = 250 (nm)
- *h*_5_ = 85 (nm)
- *w*_1_ = 300 (nm)
- *k*_1_ = *d* /*w*_1_ = 1
- *k*_2_ = *w*_2_/*d* = 0.623
- *k*_3_ = *w*_3_/*w*_2_ = 0.9
- *P* = 400 (nm)

**Oblique Incidence Configuration:**

To evaluate the absorber under oblique incidence, the plane-wave source was set to BFAST (Broadband Fixed Angle Source Technique).

- For TM polarization, the polarization angle was set to 0°, and the boundary conditions along *y-min* and *y-max* were set to symmetric.
- For TE polarization, the polarization angle was set to 90°, and the boundary conditions along *y-min* and *y-max* were set to anti-symmetric.
- Selection of the BFAST source automatically updates the remaining boundary conditions as required.

**S2. Reasons for the decrease in absorption at large angles of incidence**

As shown in Fig. 7 of the manuscript, the absorption spectrum of the proposed absorber varies with the angle of the incident light, and this behavior differs for TM and TE polarized light. The following explanation will detail the reasons for the changes in the absorption spectrum at different incident angles.

The observed reduction in absorption at very large incidence angles (greater than 60°) can be attributed to several fundamental electromagnetic mechanisms. First, oblique illumination increases the in-plane wavevector *k_x_* = *k*_0_sin*θ*. This change alters the phase matching between the incident wave and the hybridized surface and cavity modes responsible for broadband absorption. When the projection of *k*_0_sin*θ* is no longer aligned with the dispersion of these resonances, the efficiency of coupling diminishes, leading to decreased absorption ^3,4^. Second, cavity-like resonances supported by the multilayer structure must satisfy an axial phase condition given by 2*nd*cos*θ*ʹ = *m*λ. As the angle of incidence increases, the resonance wavelengths shift out of the designed band, further reducing absorption. Third, TE and TM polarizations respond differently to these angle changes. TM waves maintain a normal electric-field component, which more effectively excites surface-plasmon and magnetic-polariton modes. In contrast, TE waves do not have this capability, which explains the earlier degradation of TE absorption at high angles ^4,5^. Finally, effective impedance matching to free space, which is essential for low reflectance, is both angle- and polarization-dependent. The near-unity impedance match achieved at normal incidence deteriorates at larger angles, leading to increased reflection. All of these mechanisms stem from Maxwell’s equations and standard dispersion and phase-matching relations ^4,6^. Together, they explain why angular robustness typically has practical limits, even for well-optimized broadband absorbers.

Figs. S1 and S2 show the spatial distribution of the electric field (|*E*|) and the magnetic field (|*H*|) at the resonance wavelengths, respectively. Each figure compares the field distributions for normal incidence, as well as TM-polarized incidence at an angle of 60° and TE-polarized incidence at the same angle. These figures clearly demonstrate the independence of the LSPR and MPs with respect to the angle of the incident light.

| 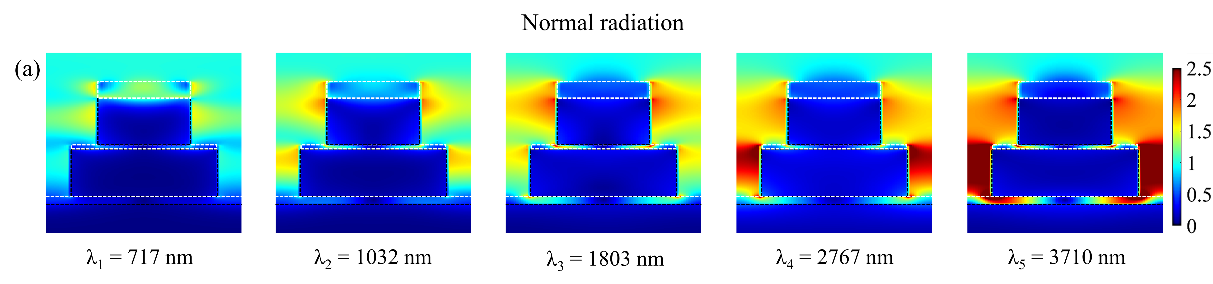 |
| --- |
| 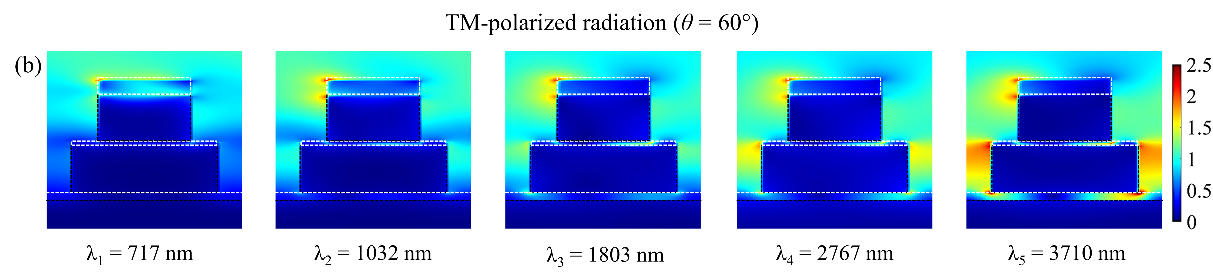 |
| 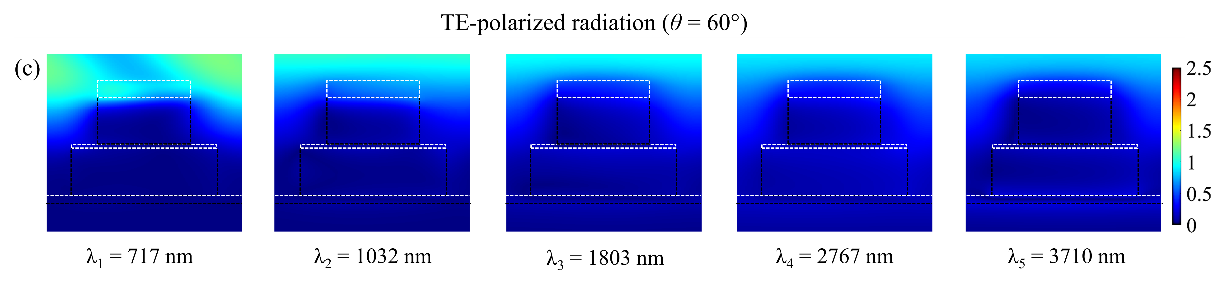 |
| Fig. S1. Spatial distribution of the electric (\|*E*\|) field intensities in the *x*-*z* plane at five resonance wavelengths under (a) normal incidence, (b) TM polarization at an angle of *θ* = 60°, and (c) TE polarization at an angle of *θ* = 60°. |

| 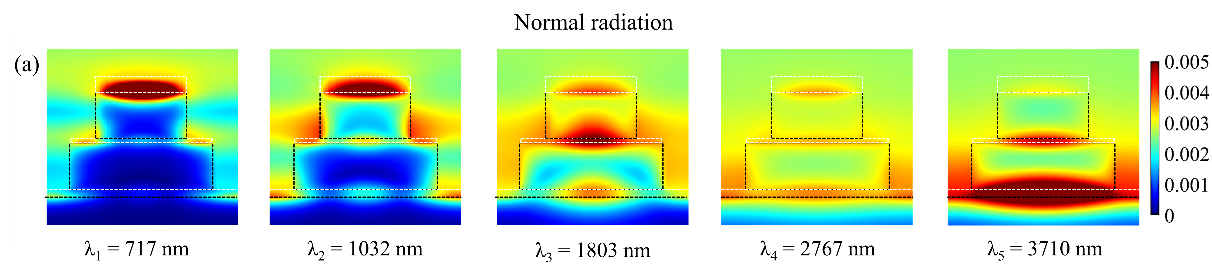 |
| --- |
| 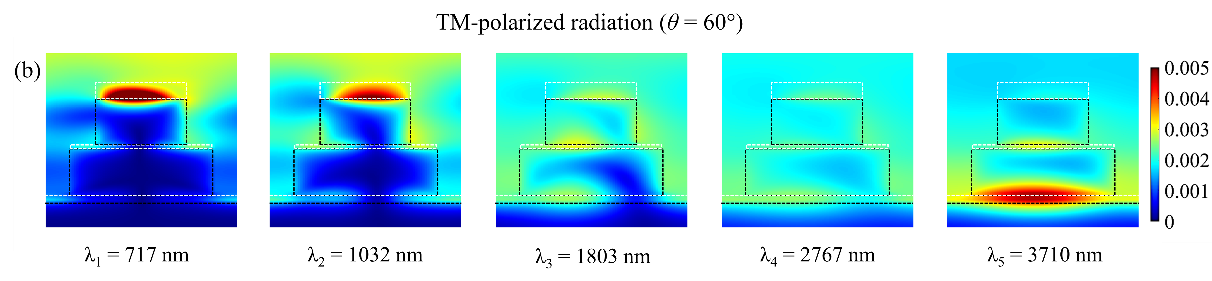 |
| 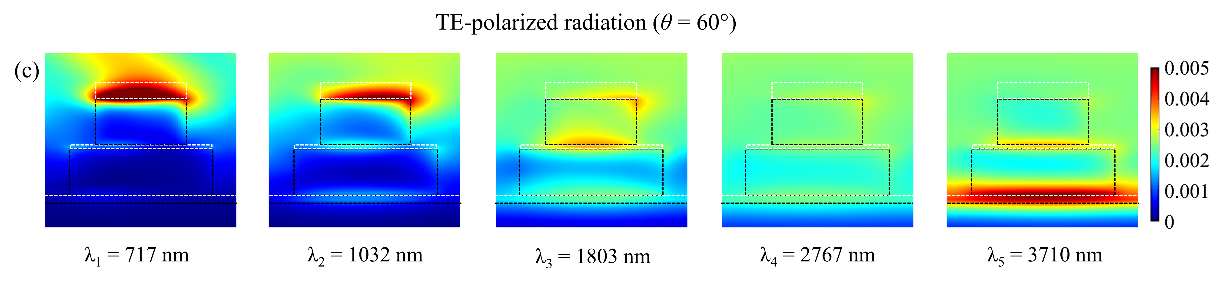 |
| Fig. S2. Spatial distribution of the magnetic (\|*H*\|) field intensities in the *x*-*z* plane at five resonance wavelengths under (a) normal incidence, (b) TM polarization at an angle of *θ* = 60°, and (c) TE polarization at an angle of *θ* = 60°. |

**References**

1 Siefke, T. *et al.* Materials pushing the application limits of wire grid polarizers further into the deep ultraviolet spectral range. *Advanced Optical Materials* **4**, 1780-1786 (2016).

2 <https://refractiveindex.info/?shelfDmain&bookDTiO2&pageDSiefke>

3 Raether, H. Plasmons on smooth and rough surfaces and on gratings. *Springer tracts in modern physics* **10** (1988).

4 Novotny, L. & Hecht, B. *Principles of nano-optics*. (Cambridge University Press, 2012).

5 Watts, C. M., Liu, X. & Padilla, W. J. Metamaterial electromagnetic wave absorbers. *Advanced materials* **24**, OP98-OP120 (2012).

6 Maier, S. A. *Plasmonics: fundamentals and applications*. Vol. 1 (Springer, 2007).
